# Interactions of Linear Analogues of Battacin with Negatively Charged Lipid Membranes

**DOI:** 10.3390/membranes11030192

**Published:** 2021-03-10

**Authors:** Kinga Burdach, Dagmara Tymecka, Aneta Urban, Robert Lasek, Dariusz Bartosik, Slawomir Sek

**Affiliations:** 1 Faculty of Chemistry, Biological and Chemical Research Centre, University of Warsaw, Żwirki i Wigury 101, 02-089 Warsaw, Poland; kburdach@chem.uw.edu.pl; 2 Faculty of Chemistry, University of Warsaw, Pasteura 1, 02-093 Warsaw, Poland; dulok@chem.uw.edu.pl (D.T.); anetaurban@student.uw.edu.pl (A.U.); 3 Faculty of Biology, Institute of Microbiology, Department of Bacterial Genetics, University of Warsaw, 02-096 Warsaw, Poland; lasek@biol.uw.edu.pl (R.L.); bartosik@biol.uw.edu.pl (D.B.)

**Keywords:** lipid membranes, battacin, antimicrobial lipopeptides, infrared spectroscopy, atomic force microscopy, Langmuir technique

## Abstract

The increasing resistance of bacteria to available antibiotics has stimulated the search for new antimicrobial compounds with less specific mechanisms of action. These include the ability to disrupt the structure of the cell membrane, which in turn leads to its damage. In this context, amphiphilic lipopeptides belong to the class of the compounds which may fulfill this requirement. In this paper, we describe two linear analogues of battacin with modified acyl chains to tune the balance between the hydrophilic and hydrophobic portion of lipopeptides. We demonstrate that both compounds display antimicrobial activity with the lowest values of minimum inhibitory concentrations found for Gram-positive pathogens. Therefore, their mechanism of action was evaluated on a molecular level using model lipid films mimicking the membrane of Gram-positive bacteria. The surface pressure measurements revealed that both lipopeptides show ability to bind and incorporate into the lipid monolayers, resulting in decreased ordering of lipids and membrane fluidization. Atomic force microscopy (AFM) imaging demonstrated that the exposure of the model bilayers to lipopeptides leads to a transition from the ordered gel phase to disordered liquid crystalline phase. This observation was confirmed by attenuated total reflection Fourier-transform infrared spectroscopy (ATR-FTIR) results, which revealed that lipopeptide action causes a substantial increase in the average tilt angle of lipid acyl chains with respect to the surface normal to compensate for lipopeptide insertion into the membrane. Moreover, the peptide moieties in both molecules do not adopt any well-defined secondary structure upon binding with the lipid membrane. It was also observed that a small difference in the structure of a lipophilic chain, altering the balance between hydrophobic and hydrophilic portion of the molecules, results in different insertion depth of the active compounds.

## 1. Introduction

The resistance of pathogens to available antibiotics is an increasingly serious problem in modern medicine [1]. This necessitates the search for new drugs which would exhibit different mechanisms of bactericidal activity compared with typical antibiotics acting on specific biochemical processes. A possible solution to this problem involves the use of compounds with less specific action, based, for example, on damaging the bacterial cell membrane. This condition is met by antimicrobial peptides targeting the bacterial cell membranes [2,3]. Most often, antimicrobial peptides are positively charged. As a result, they interact preferentially with negatively charged membranes of bacterial cells, and their amphiphilic structure allows them to insert into the core of the membrane [4]. Such a model of action is widely accepted since the bactericidal kinetics of antimicrobial peptides is often correlated with the depolarization of the cell membrane. This shows that the mode of action involves disruption of the membrane integrity. Possible mechanisms include pore formation or membrane solubilization in a detergent-like manner [5]. A similar mode of action can be expected for short lipopeptides containing an acyl chain usually coupled to the N-terminus of a peptide moiety, which includes 2–8 amino-acid residues [6]. The advantage of short lipopeptides lies in their simpler structure and, therefore, easier synthesis, as well as in the wide possibilities for modifying their structure in terms of the balance between the hydrophilic and hydrophobic parts, as well as the charge density and distribution. For that reason, lipopeptides seem to be suitable for drug design, and some of them are already approved for clinical use. Examples include daptomycin and polymyxins [7,8].

Battacin is an example of a cationic cyclic lipopeptide, and it is isolated from bacterium *Paenibacillus tianmuensis* [9]. Like other lipopeptides, it consists of two parts: a lipophilic chain containing 3-hydroxy-6-methyloctanoic acid attached to the peptide composed of eight D- and L-amino acids, where seven of them form a ring. Battacin has noncoded amino acid α,γ-diaminobutyric acid (Dab) in both D- and L- forms, which provide resistance to proteases. It was found to exhibit bactericidal activity against Gram-negative and Gram-positive bacteria, including multidrug-resistant and extremely drug-resistant clinical isolates. Unfortunately, in naturally occurring battacin, high efficacy against multidrug-resistant bacterial strains is accompanied by nephro- and neurotoxicity, which eliminate its use as a clinical drug. To overcome this problem, numerous derivatives of battacin were designed and synthesized [10,11,12]. Among them, a promising class includes linear analogues, which were demonstrated to be active in terms of lysing bacteria and dispersing biofilms. Recently, the results of molecular dynamics simulations were reported by Chakraborty and coworkers, which shed some light on the possible action mechanism of battacin analogues [13]. It was demonstrated that the activity of linear analogues of battacin depends on the balance between the positively charged and hydrophobic moieties. It was found that the hydrocarbon chain of the lipidated N-terminal residue and the hydrophobic amino-acid residues, i.e., D-Phe and Leu, insert into the membrane core and anchor the lipopeptide to the membrane. The presence of Dab residues improves membrane binding through electrostatic interactions and increased hydrogen bond formation. The interesting feature of these compounds is that, unlike typical antimicrobial peptides, their activity is not based on the presence of a specific secondary structure when bound to a lipid membrane. Hence, the mechanism of their membranolytic action may differ from those observed for antimicrobial peptides.

**Scheme 1:**
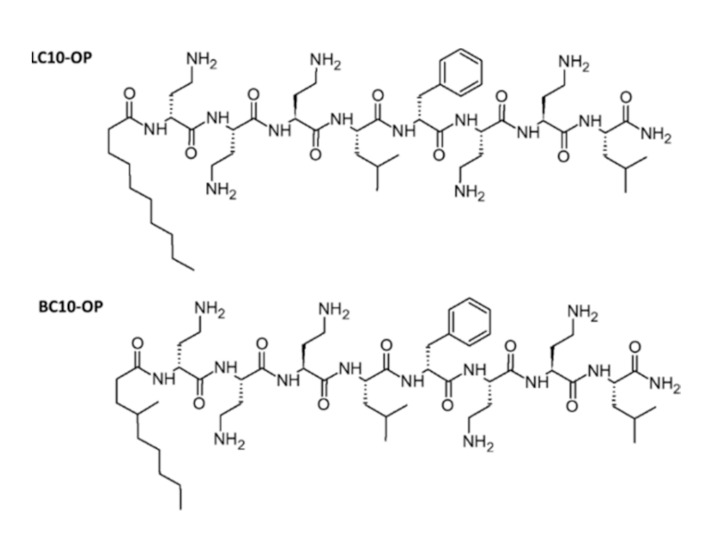
Chemical structure of lipopeptides.

In this paper, we characterized two linear analogues of battacin with a peptide moiety containing the same sequence of amino acids as natural battacin, but the lipophilic chain composed of 3-hydroxy-6-methyloctanoyl was replaced either with a linear decanoyl chain (LC10-OP) or with a branched 4-methylnonanoyl chain (BC10-OP). These lipophilic chains were chosen to modulate the balance between the hydrophobic and hydrophilic portion of lipopeptides, which may affect their ability to insert into the lipid membrane. As demonstrated by Neubauer and coworkers, acyl chain branching in short lipopeptides makes them more hydrophilic compared with the analogues possessing the same number of carbon atoms [14]. Moreover, the same authors observed that short lipopeptides with a branched fatty acid chain cause distinctly lower hemolysis compared with the reference lipopeptides with similar hydrophobicity or the same number of carbon atoms in a linear hydrocarbon chain. The chemical structures of the lipopeptides studied in this work are shown in Scheme 1.

The lipopeptides were tested in terms of their antimicrobial activity, and the mechanism of their action was evaluated on a molecular level using model lipid films, i.e., Langmuir monolayers and solid-supported lipid bilayers. The physicochemical characterization of lipopeptide–membrane interactions was performed using surface-sensitive techniques including surface pressure measurements, atomic force microscopy (AFM), and attenuated total reflection Fourier-transform infrared spectroscopy (ATR-FTIR). These methods enabled evaluation of the lipopeptide-induced changes in the structure of a model lipid membrane.

## 2. Materials and Methods 

### 2.1. Chemicals

1-Palmitoyl-2-oleoyl-*sn*-glycero-3-phosphoglycerol (POPG), 1,2-dipalmitoyl-*sn*-glycero-3-phosphoglycerol (DPPG), and 1′,3′-bis[1,2-dimyristoyl-*sn*-glycero-3-phospho]-glycerol (CL) were purchased from Avanti Polar Lipids Inc. Ultrapure methanol and chloroform, which were used to dissolve lipids, were purchased from Sigma Aldrich. Sodium chloride, sodium phosphate, potassium chloride, and potassium phosphate were purchased from Avantor Performance Materials Poland S.A. Analytical-grade sodium hydroxide and hydrochloric acid were obtained from Chempur. These reagents were used to prepare phosphate-buffered saline (abbreviated as PBS). The aqueous solution of PBS (10 mM) was adjusted to pH = 7.4. Stock solutions of lipopetides and the aqueous buffer solution of PBS were prepared with Milli-Q water (final resistivity 18.2 MΩ·cm).

### 2.2. Synthesis of Lipopeptides

The LC10-OP and BC10-OP lipopeptides were synthesized using the well-established standard Fmoc/t-Bu methodology used for solid-phase peptide synthesis [15]. An analogous protocol of lipopeptide synthesis was recently reported by our group [16]. Rink Amide AM resin (substitution level of 0.55 mmol/g) and Fmoc-protected amino-acid building blocks in a standard Fmoc-Xaa-OH/TBTU/DIPEA protocol (2 eq/2 eq/4 eq) were used. The same protocol was used for the fatty-acid coupling to the N-termini of the peptide moiety. The final lipopeptide was cleaved from the resin using Reagent B (trifluoroacetic acid/phenol/H_2_O/triisopropyl silane 88:5:5:2; *v*/*v*/*v*/*v*). The final compounds were purified using reverse-phase HPLC (Shimadzu Prominence system) and C18 Luna column (Phenomenex, 150 mm× 10 mm, 5 μm) with a linear gradient of H_2_O–acetonitrile–0.1% TFA, and the relevant fractions were lyophilized. The purity of LC10-OP and BC10-OP was assessed from analytical HPLC, and it was higher than 95%. The retention times for LC10-OP and BC10-OP were 26.4 min and 25.7 min, respectively. Because the lipopeptides were to be subjected to biological tests, it was necessary to replace the trifluoroacetate counterion with the chloride counterion. The lipopeptides were analyzed by mass spectrometry and FTIR. High-resolution electrospray ionization mass spectrometry (HR ESI-MS): LC10-OP [M + 2H]^2+^ *m*/*z* = 523.36, [M + 3H]^3+^ *m*/*z* = 349.24; BC10-OP [M + 2H]^2+^ *m*/*z* = 523.36, [M + 3H]^3+^ *m*/*z* = 349.24. FTIR (KBr): LC10-OP ν 3431, 3331, 3031, 2960, 2928, 1671, 1633, 1530, 1512, 1470, 1397, 1340, 1280, 1229, 1167 cm^−1^; BC10-OP ν 3434, 3329, 3031, 2959, 2928, 1674, 1661, 1633, 1530, 1512, 1470, 1399, 1338, 1277, 1226, 1170 cm^−1^.

### 2.3. Biological Activity

The bacterial strains were acquired from the Polish Collection of Microorganisms (PCM) or American Type Culture Collection (ATCC). Gram-positive strains were as follows: *Staphylococcus aureus* ATCC 29213, *Staphylococcus epidermidis* ATCC 12228, and *Enterococcus faecalis* ATCC 14506. Gram-negative strains were as follows: *Pseudomonas aeruginosa* PAO1 PCM 499, *Klebsiella pneumoniae* PCM 1, and *Yersinia enterocolitica* PCM 2081. The strains were grown in lysogeny broth (LB). Single-colony material was transferred to 10 mL of LB medium and grown at 30 °C with shaking overnight. The optical density of the overnight cultures (at 600 nm) was then adjusted to 0.05 by dilution with a fresh portion of LB medium. The lipopeptides were dissolved in water. Serial dilutions of compounds were prepared in LB medium ranging from 5 to 50 mg/L (final concentrations). The experiment was performed by adding 100 µL of each of the prepared dilutions to 100 µL of the diluted overnight culture of bacteria in the wells of a 96-well microtiter plate. The minimum inhibitory concentration (MIC) was defined as the lowest concentration of test compound needed to inhibit bacterial growth when evaluated after 24 h incubation at 30 °C with shaking (final optical density at 600 nm not greater than 0.05). Optical density measurements were performed using a TECAN Sunrise plate reader. Data were obtained from three independent experiments.

### 2.4. Surface Pressure Measurements

Lipid monolayers at the air/buffer interface were formed using a KSV NIMA Langmuir trough (Biolin Scientific, Sweden) equipped with two movable hydrophilic barriers. A Wilhelmy plate made of filter paper was used to measure the surface pressure. Before each experiment, the barriers and the trough were cleaned with a chloroform/methanol mixture and then Milli-Q water. Lipopeptides LC10-OP and BC10-OP were dissolved in water. POPG was dissolved in a chloroform, DPPG was dissolved in a chloroform/methanol (65:35, *v*/*v*) mixture, and CL was dissolved in a chloroform/methanol (4:1, *v*/*v*) mixture. After preparation of lipid solutions, the stock solution of the DPPG/POPG/CL (1:1:2 mol/mol/mol) mixture was prepared with final concentration of 1 mg/mL. All lipid monolayers were formed on an aqueous solution of 0.01 M phosphate buffer saline (pH 7.4) either without or with addition of lipopeptides. The lipid mixture was applied onto the subphase using a Hamilton syringe (50 µL). Spreading solutions were left on buffer subphase for 10 min to complete solvent evaporation. Monolayers were compressed at the barriers speed of 10 mm/min and at constant temperature 21 ± 1 °C to record the surface pressure versus molecular area isotherms. All measurements were repeated at least three times to ensure reproducible results.

### 2.5. Preparation of Small Unilamellar Vesicles

Stock solutions containing ∼5.0 mg/mL of the desired lipids were prepared in a similar way as for surface pressure measurements. Then the solutions were mixed in a test tube at the desired molar ratio (DPPG/POPG/CL 1:1:2). Next, the solvent was evaporated by vortexing under a stream of nitrogen, and the test tube containing dried lipid cake was placed in a vacuum desiccator for 1 h to remove the residues of solvent. Then, 1.0 mL of an aqueous solution of 0.01 M PBS was added to the lipid cake, and the mixture was sonicated at ∼40 °C for 1 h. For infrared measurements, D_2_O was used for buffer preparation. After the sonication step, the suspension of lipid vesicles was homogeneous and transparent.

### 2.6. Atomic Force Microscopy

Atomic force microscopy (AFM) experiments were carried out with Dimension Icon (Bruker) in PeakForce Tapping Mode with simultaneously recorded nanomechanical data including Young’s modulus. ScanAsyst Fluid probes (Bruker, nominal spring constant 0.7 N/m) were used for imaging. The exact value of the spring constant was obtained by thermal tune method before each experiment. Lipid bilayers were deposited on freshly cleaved mica substrates by vesicle spreading. The samples were imaged under in situ conditions in an aqueous 0.01 M PBS solution at the temperature of 21 ± 1 °C.

### 2.7. Attenuated Total Reflection Fourier-Transform Infrared Spectroscopy

All spectra were collected using a Nicolet iS50 infrared spectrometer (Thermo Fisher Scientific Inc.) equipped with a liquid-nitrogen-cooled MCT-A detector and custom-made single-reflection accessory. The incident angle was set at 55°, and the spectral resolution was 4 cm^−1^. The spectra are presented in absorbance units A = log(*I*_0_/*I*), where *I*_0_ and *I* correspond to the single-beam intensities of IR radiation observed for the reference and the sample, respectively. In all experiments, we used a silicon hemispherical prism. The bilayer was deposited on the planar surface of the prism by spreading of lipid vesicles. The refractive indices used for the molecular orientation calculations were 3.42 for Si, 1.45 for lipid bilayer, and 1.32 for D_2_O. Data processing was performed with Omnic 9 software (Thermo Fisher Scientific Inc., Waltham, MA, USA).

## 3. Results and Discussion

Lipopeptides LC10-OP and BC10-OP were first tested for their potential antimicrobial activity. For that purpose, we determined their minimum inhibitory concentrations (MICs), which is the lowest concentration of a compound which prevents visible growth of bacteria. The values of MIC for both lipopeptides against selected strains of bacteria are shown in Table 1.

The results of the measurements summarized in Table 1 show that both lipopeptides exhibited variable activity against the tested bacterial strains. Their activity against Gram-negative strains *Y. enterocolitica* and *K. pneumoniae* was found to be relatively low. The same applied to Gram-positive *E. faecalis*. Noticeably higher activity was observed against Gram-negative *P. aeruginosa*. However, the lowest values of MIC and, hence, the highest activity were found for Gram-positive *S. aureus* and *S. epidermidis*, which may suggest a certain degree of selectivity of the lipopeptides against these strains. Because the tested lipopeptides are amphiphilic in nature, it can be assumed that their antimicrobial activity is based on the interaction with the bacterial cell membrane. To verify this hypothesis, a physicochemical characterization of lipopeptides interactions with lipid membranes was performed. Due to their high activity against *S. aureus* and *S. epidermidis*, the composition of the model films was selected to mimic the lipid composition of the cell membranes of Gram-positive bacteria [17].

**Figure 1:**
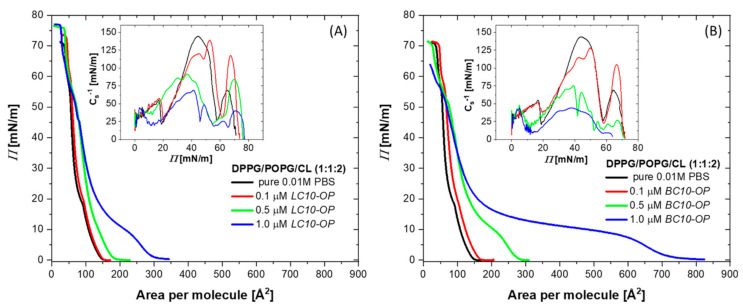
Surface pressure (*Π*) versus area per molecule (*A*) isotherms recorded for 1-palmitoyl-2-oleoyl-*sn*-glycero-3-phosphoglycerol (POPG), 1,2-dipalmitoyl-*sn*-glycero-3-phosphoglycerol (DPPG), and 1′,3′-bis[1,2-dimyristoyl-*sn*-glycero-3-phospho]-glycerol (CL) monolayers in the absence (black curves) and in the presence of lipopeptides dissolved in a subphase (colored curves). Panel (**A**) demonstrates the results for different concentrations of LC10-OP, while panel (**B**) shows the results obtained for BC10-OP. An aqueous solution of 0.01 M phosphate-buffered saline (PBS) was used as a subphase. The insets show the changes in the compression modulus as a function of surface pressure.

Initially, the effect of LC10-OP and BC10-OP lipopeptides on model bacterial lipid membranes was studied using the Langmuir technique. As a model, we utilized a negatively charged lipid membrane composed of DPPG/POPG/CL (1:1:2). Lipopeptides were dissolved in an aqueous 0.01 M PBS subphase, and their final concentration was varied from 0.1 µM to 1 µM. The results are shown in Figure 1. Initially, the lipid monolayers were compressed on PBS subphase without lipopeptides. The surface pressure (*Π*) vs. area per molecule (*A*) isotherms of DPPG/POPG/CL monolayer display the lift-off at molecular areas of ~150 Å^2^, and, at the surface pressure of ~18 mN/m, the phase transition from a liquid-expanded to a liquid-condensed phase appeared. The partial collapse of the monolayer occurred at ~56 mN/m. This is related to the collapse of POPG molecules, which are squeezed out from the monolayer [18]. The removal of POPG increases the condensation of the monolayer, and the mixture of DPPG and cardiolipin is further compressed up to ~70 mN/m, where the second collapse occurs. Because the aim of the experiment was to examine the effect of lipopeptides on a three-component monolayer, the data recorded above the POPG collapse were not analyzed. The introduction of lipopeptides into the subphase shifted the DPPG/POPG/CL isotherm toward larger molecular areas, and the effect was noticeable even at the lowest concentration of lipopeptides. Such behavior indicates that lipopeptides were incorporated into the DPPG/POPG/CL monolayer, and the effect was better pronounced at the beginning of the monolayer compression. However, the lift-off of the isotherms recorded on the subphase containing BC10-OP started at a larger molecular area compared with LC10-OP, which shows that, at low surface pressure, BC10-OP was incorporated more easily into DPPG/POPG/CL membrane.

A more quantitative analysis of lipopeptide incorporation into the lipid monolayers can be performed on the basis of limiting molecular area values determined from the *Π*–*A* isotherms. The results collected in Table 2 clearly show that the area per molecule grew with the increasing concentration of the lipopeptide in the subphase, which can be ascribed to the increasing number of molecules incorporated from the subphase [19,20]. The general trend was the same for both compounds, but the increase in the area per molecule was significantly greater for BC10-OP. For example, the values of the limiting molecular area determined for monolayers compressed on buffer with 1 μM concentration of lipopeptides were found to be ~142 Å and ~181 Å for LC10-OP and BC10-OP, respectively. This demonstrates the enhanced ability of BC10-OP to be incorporated into the lipid monolayer compared to LC10-OP. Another important aspect of the properties of lipid monolayers can be obtained from the value of the compression modulus (*C*_s_^−1^), which is defined as follows [21]:

(1) $$C_{s}^{-1}=\;-A\frac{d\Pi }{dA}$$

where *Π* is a surface pressure and *A* is the area per molecule. This parameter provides information on the state in which the monolayer exists at a given surface pressure. It is widely accepted that a compression modulus in the range of 12.5–100 mN/m corresponds to the liquid expanded state, 100–250 mN/m is characteristic of a liquid condensed state, and values above 250 mN/m are indicative of a solid state. The maximum value of compression modulus for the DPPG/POPG/CL monolayer compressed on pure buffer subphase was 139 mN/m, which means that the monolayer was in a liquid-condensed state (see Figure 1). The addition of lipopeptides at the lowest concentration had no significant effect on the value of the compression modulus. However, at the concentration of 0.5 μM, a significant drop was already observed; finally, at the lipopeptide concentration of 1 μM, the values of *C*_s_^−1^ were found to be 70 mN/m and 54 mN/m for LC10-OP and BC10-OP, respectively. This demonstrates that the monolayers existed in a liquid expanded state. Hence, the incorporation of lipopeptides decreased the molecular packing density within the lipid film and caused monolayer fluidization. However, the fluidizing effect of BC10-OP was stronger compared with LC10-OP as can be deduced from the data collected in Table 2.

**Figure 2:**
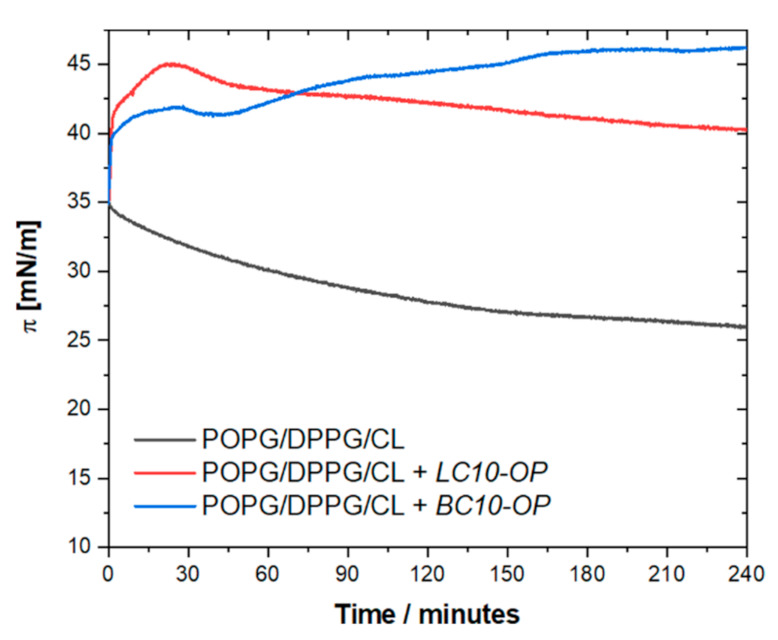
Changes in surface pressure as a function of time recorded for DPPG/POPG/CL monolayer compressed to initial surface pressure of 35 mN/m (black curve) and DPPG/POPG/CL monolayers compressed to initial surface pressure of 35 mN/m after injection of LC10-OP (red curve) and BC10-OP (blue curve) into the subphase. An aqueous solution of 0.01 M PBS was used as a subphase.

Since, under biological conditions, antimicrobial substances interact with already existing cell membranes, we investigated the effect of lipopeptides on monolayers pre-formed at the air–buffer interface. For that purpose, the lipid monolayers were compressed to 35 mN/m. This value of the surface pressure was chosen to achieve the structural organization of the lipid film resembling that in natural cell membranes. After monolayer compression, the position of the barriers of the Langmuir trough was fixed to maintain a constant area occupied by the lipid film. Furthermore, a stock solution of either LC10-OP or BC10-OP lipopeptide was injected into the subphase under the film to reach a final concentration of 1 µM. Then, the changes in the surface pressure were monitored as a function of time (see Figure 2).

The surface pressure of the DPPG/POPG/CL monolayer measured in the absence of lipopeptides decreased slightly over time, which may be related to the partial solubility of the lipids in the subphase. Additionally, all lipid components were negatively charged, and the monolayer at 35 mN/m existed in a densely packed and ordered state; hence, the repulsive forces between polar heads may have contributed to the expulsion of some molecules into the bulk of the subphase solution. The injection of lipopeptides resulted in a rapid increase in surface pressure. An analysis of the slope of the curves during the first few minutes upon injection revealed that, initially, the kinetics of binding was similar for both lipopeptides. This is reasonable since, at the initial stage, the interactions were mostly driven by electrostatic attractions between the positively charged peptide moiety, which was the same in both lipopeptides, and the negatively charged lipid polar headgroups. Nevertheless, the analysis of the curves after longer times demonstrated that the lipopeptides showed noticeable differences in behavior. In the presence of LC10-OP, after the initial step of binding to the lipid film, the surface pressure curve reached a maximum and then started to decline gently. For BC10-OP, the surface pressure gradually increased until it reached a quasi-equilibrium of about 45 mN/m. This observed behavior may have been the result of slight differences in the action of lipopeptides. We can assume that LC10-OP electrostatically interacts with negatively charged lipids, but this interaction is not counterbalanced by hydrophobic interactions driving the lipopeptide insertion. Therefore, a substantial fraction of the lipopeptide molecules remains in the region of the polar heads. This prevents further accumulation of the lipopeptides and only a small fraction anchors the lipophilic tail in the monolayer. Electrostatic interactions occur also between BC10-OP and lipid polar heads; however, in this case, the barrier for the reorientation and incorporation of the lipopeptide into the lipid film is smaller, such that a larger fraction of molecules can insert between the lipid chains.

**Figure 3:**
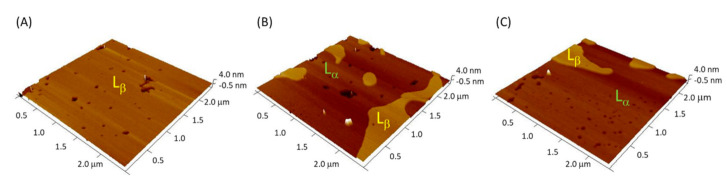
Atomic force microscopy (AFM) images collected for DPPG/POPG/CL bilayer deposited on mica before (**A**) and after ~30 min of the exposure to LC10-OP (**B**) and BC10-OP (**C**). The images were recorded in aqueous 0.01 M PBS.

Although Langmuir monolayers are widely accepted model systems, they do not reproduce the bilayer architecture of cell membranes. Therefore, the membranolytic properties of lipopeptides were further investigated with solid-supported lipid bilayers [22]. Such bilayers are certainly a better model of the natural cell membranes. Nevertheless, they also have some limitations due to the interaction of lipid molecules with the substrate, which may, for example, affect the hydration of the polar heads in the bottom leaflet. This effect is often minimized using hydrophilic substrates such as mica, glass, or quartz. To evaluate the changes in topography and thickness of the DPPG/POPG/CL bilayer upon exposure to lipopeptides, we performed AFM experiments. This technique enables mesoscale imaging of the surface structures under in situ conditions; hence, it is possible to follow the dynamics of numerous surface-related processes [23,24]. The bilayers were deposited on the mica surface by spreading small unilamellar vesicles. The AFM images of the resulting DPPG/POPG/CL bilayer before and after exposure to lipopeptides are illustrated in Figure 3.

The bilayers were analyzed in terms of the lipopeptide-induced changes in their topography and thickness. The latter was determined on the basis of cross-sectional profiles taken along the defect sites, and it was calculated as the average height difference between the bare substrate and the region covered by the lipid membrane. This approach is characterized by simplicity, but it should be noted that the obtained thickness of the lipid layers may be slightly underestimated due to the elastic deformation of the membrane under the load of the AFM probe. Thus, the obtained thickness may be slightly lower compared with equilibrium conditions. As demonstrated in Figure 3A, the average thickness of the intact DPPG/POPG/CL membrane was found to be 5.0 ± 0.2 nm. As a function of the value of the Young’s modulus, which was determined to be ~29 MPa, it can be concluded that the bilayer existed mostly in the gel (L_β_) phase [25]. After the injection of the lipopeptides, the morphology of the films changed noticeably. In both cases, the effect of the membrane thinning was observed, and the Young’s modulus determined in topographically lower regions was ~16 MPa. This reflects a lipopeptide-induced disordering effect and the transition from the gel L_β_ phase to liquid crystalline L_α_ phase [25]. After approximately 30 min of exposure, both phases coexisted; however, the L_β_ phase domains were reduced to 10–20% of the scanned area. The thickness of the DPPG/POPG/CL bilayer in the L_α_ phase region was determined to be 4.0 ± 0.3 nm and 3.9 ± 0.4 nm for LC10-OP and BC10-OP, respectively. The AFM data indicate clearly that the interactions of lipopeptides with the supported lipid bilayer resulted in a decreased ordering of the lipids and led to the fluidization of the membrane. A similar effect was recently reported by our group for short amphiphilic lipopeptides with a general structure of C_n_-fXXL, where *n* = 12, 14, or 16 and X denotes the Dab residue [16]. The reduction in bilayer thickness can be explained in terms of Israelachvili′s concept of the critical packing parameter (cpp) [26,27,28]. This parameter is defined as the ratio between the hydrocarbon tail effective area and the projection area of the polar peptide headgroup. For lipid bilayers, the values of cpp are usually between 1/2 and 1. By using the additivity of the cpp, the weighted average value determined for the DPPG/POPG/CL bilayer was close to unity. The binding and partitioning of the lipopeptides is expected to lower the additive value of cpp. This is related to the conical shape of lipopeptides resulting from the large size of the polar head (i.e., the value of cpp for lipopeptides is expected to be ∼1/3). In order to compensate for the presence of the large polar heads, lipid molecules increase their tilt angle with respect to the surface normal, and the intermolecular distances are also increased. Consequently, the bilayer accommodating the lipopeptides becomes thinner and more fluid-like. However, due to the structural variation of the lipophilic chains in lipopeptides (i.e., linear in LC10-OP vs. branched in BC10-OP), the exact titling of lipid molecules might be slightly different.

Quantitative information regarding the structure of the bilayers before and after lipopeptides binding was obtained from ATR-FTIR measurements. This enabled the analysis of the orientation and ordering of lipid molecules within the membrane. The orientation of the molecules assembled on planar surface of the Si prism can be determined from polarized ATR-FTIR spectra since the intensity of the IR band depends on the angle between the vectors of the transition dipole moment of given vibration and the electric field of the incident beam. To determine the molecular orientation, one needs to obtain the information about the direction and the amplitude of the electric field at the interface. To control the direction of the electric field, linearly polarized light can be used. For the penetration depth of the evanescent wave greatly exceeding the thickness of the lipidic assembly, a thin film approximation can be applied, and the amplitudes of spatial components of the electric field (*E*_x_, *E*_y_, *E*_z_) may be calculated using following equations [29]:

(2) $$E_{x}=\frac{2\cos\theta_{1}{\left(sin^{2}\;\theta_{1}-n_{31}^{2}\right)}^{1/2}}{{\left(1-n_{31}^{2}\right)}^{1/2}{\left[\left(1+n_{31}^{2}\right)sin^{2}\;\theta_{1}-n_{31}^{2}\;\right]}^{1/2}}\;E_{y}=\frac{2\cos\theta_{1}}{{\left(1-n_{31}^{2}\right)}^{1/2}}$$

(3) $$E_{z}=\frac{2n_{32}^{2}\sin\theta_{1}\cos\theta_{1}}{{\left(1-n_{31}^{2}\right)}^{1/2}{\left[\left(1+n_{31}^{2}\right)sin^{2}\;\theta_{1}-n_{31}^{2}\;\right]}^{1/2}}$$

where *θ*_1_ denotes the angle of incidence of IR beam at the solid–liquid interface; *n*_32_ = *n*_3_/*n*_1_ and *n*_32_ = *n*_3_/*n*_2_, where *n*_1_, *n*_2_, and *n*_3_ are refractive indices of the internal reflection element (Si prism), thin film (lipid membrane), and bulk medium (aqueous solution), respectively. In the case of the lipid bilayer deposited on the planar surface of an Si prism, the dichroic ratios (*R*) can be determined experimentally as the ratio of the absorbance of p-polarized and s-polarized light. Once the dichroic ratio and the electric field amplitudes of the evanescent wave are known, it is possible to calculate the order parameter (*S*_dipole_) and orientation angle (*θ*_dipole_) with respect to the surface normal for given transition dipole moment [30,31].

(4) $$S_{dipole}=\frac{E_{x}^{2}-RE_{y}^{2}+E_{z}^{2}}{E_{x}^{2}-RE_{y}^{2}-2E_{z}^{2}}$$

(5) $$\theta_{dipole}=cos^{-1}\sqrt{\frac{2S_{dipole}+1}{3}}$$

If the structure of the molecules forming the film is well defined, there is a strict relationship between the direction of the transition dipole moment of given vibration and the molecular axis. In the case of lipids, the transition dipole moments of υ_s_(CH_2_) and υ_as_(CH_2_) are oriented perpendicular (*α* = 90°) to the molecular axis defined by trans segments of hydrocarbon chains. Hence, using these bands, it is possible to estimate the average tilt angle of the acyl chains (*θ*_chain_) with respect to the surface normal.

(6) $$S_{chain}=\frac{E_{x}^{2}-RE_{y}^{2}+E_{z}^{2}}{\frac{1}{2}\left(3cos^{2}\alpha -1\right)\;(E_{x}^{2}-RE_{y}^{2}-2E_{z}^{2})}$$

(7) $$\theta_{chain}=cos^{-1}\sqrt{\frac{2S_{chain}+1}{3}}$$

**Figure 4:**
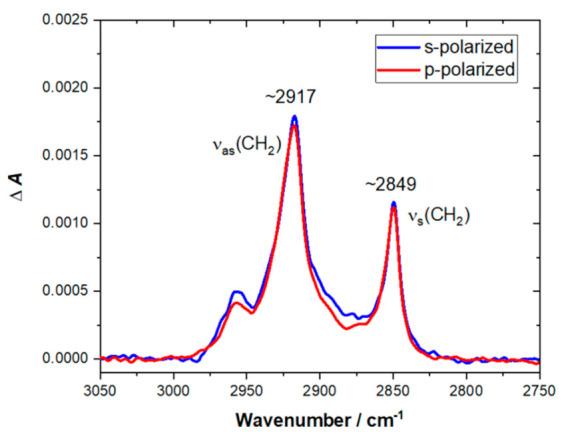
C–H stretching region of attenuated total reflection Fourier-transform infrared spectroscopy (ATR-FTIR) spectra recorded for DPPG/POPG/CL bilayer on Si prism after 90 min of lipid film deposition and washing the cell with pure buffer solution. The spectra were recorded in 0.01 M PBS dissolved in D_2_O. The red line corresponds to the spectrum recorded with p-polarized light, while the blue line corresponds to the spectrum recorded with s-polarized light.

Under the experimental conditions used in this work, the penetration depth of the evanescent wave at the wavelength corresponding to the C–H stretching region was expected to be ~0.20 μm, while the thickness of the bilayer was ~5.0 nm. This means that the thin film approximation could be safely applied to determine molecular orientation. The successful formation of the DPPG/POPG/CL lipid membrane on the planar surface of the hemispherical Si prism was confirmed by ATR-FTIR spectra. An increase in positive bands in the C–H stretching region was observed in time, and, after approximately 60–90 min, the intensity of the bands did not change. The spectra shown in Figure 4 were recorded after approximately 90 min of lipid film deposition upon gently washing the cell with pure buffer to remove excess liposomes. The spectrum of the bare Si prism recorded in 0.01 M PBS was used as a reference.

The position of υ_as_(CH_2_) and υ_s_(CH_2_) bands enables drawing a conclusion about the physical state and packing density of the acyl chains in lipid membrane. The frequencies lower than ~2920 cm^−1^ for the υ_as_(CH_2_) band and lower than ~2850 cm^−1^ for the υ_s_(CH_2_) band are characteristic for the ordered gel state of a bilayer with fully stretched acyl chains [31,32]. Higher frequencies of υ_as_(CH_2_) and υ_s_(CH_2_) bands are indicative of an increasing number of gauche defects and disordering. Hence, for the disordered liquid crystalline state, the υ_as_(CH_2_) and υ_s_(CH_2_) bands can be shifted up to ~2924 cm^−1^ and ~2853 cm^−1^, respectively. As shown in Figure 4, the υ_as_(CH_2_) band was located at 2917 cm^−1^, and the υ_s_(CH_2_) band appeared at 2849 cm^−1^, which proved that the acyl chains were ordered and the membrane was in a gel state. Since the spectra were recorded for both p- and s-polarization, it was possible to calculate the dichroic ratio, which was found to be 0.96 (±0.02) for the υ_s_(CH_2_) band. The resulting value of the order parameter for the acyl chain (*S*_chain_) was 0.78 (±0.03), which was also indicative of the gel state. The average tilt angle of the acyl chains with respect to the surface normal (*θ*_chain_) was determined to be 22° ± 2°.

**Figure 5:**
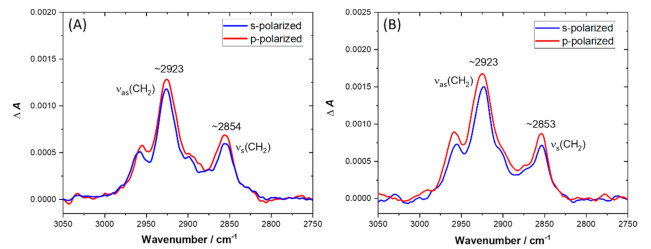
C–H stretching region of ATR-FTIR spectra recorded for DPPG/POPG/CL bilayer deposited onto the Si prisms after ~45 min of exposure to LC10-OP (**A**) and BC10-OP (**B**). The spectra were recorded in 0.01 M PBS dissolved in D_2_O. The red line corresponds to the spectrum recorded with p-polarized light, while the blue line corresponds to the spectrum recorded with s-polarized light.

Furthermore, the DPPG/POPG/CL bilayers were exposed to lipopeptides. In this case, the spectra of the intact lipid bilayers in the absence of lipopeptides were used as the reference, and the changes in absorption were again monitored in time. The binding of the lipopeptides to lipid bilayers caused the emergence of absorption bands within the (C–H) stretching region, and their intensity gradually increased up to 30–45 min before achieving the steady state. Figure 5 shows the resulting spectra in the C–H stretching region recorded after ~45 min of exposure.

In a control experiment without the lipid membrane, we found that the contribution of hydrocarbon chains from lipopeptides to the absorption spectra was negligible. Therefore, the observed growth of the intensity of (C–H) stretching bands could be interpreted as an increase in the tilt angle of acyl chains with respect to the surface normal. The direction of the (C–H) stretching vibrations was perpendicular to the axis of the acyl chain; hence, the increased tilt angle of lipid molecules caused the enlargement of the transition dipole moment component parallel to the surface normal. Interestingly, the υ_as_(CH_2_) and υ_s_(CH_2_) bands were located at ~2923 cm^−1^ and ~2853 cm^−1^, demonstrating that the ordering of lipids was affected by the presence of lipopeptides, and the bilayers existed in a disordered liquid crystalline state. To obtain the quantitative information on the orientation of lipids upon exposure to lipopeptides, we performed more detailed analysis of the p- and s-polarized spectra. The relevant parameters including dichroic ratios (*R*), order parameters (*S*_chain_), and tilt angles (*θ*_chain_), extracted from the p- and s-polarized spectra of DPPG/POPG/CL bilayers before and after lipopeptide binding, are collected in Table 3.

In the case of the bilayer exposed to LC10-OP, the order parameter was 0.50 (±0.06) and the acyl chain tilt angle with respect to the surface normal was 35° (±2°). The values of order parameter and the tilt angle determined for the bilayer exposed to BC10-OP were 0.43 (±0.04) and 38° (±1°), respectively. Hence, in both cases, the order parameters decreased and the tilt angles with respect the surface normal increased compared with the intact DPPG/POPG/CL bilayer. These results show that lipopeptide binding decreased ordering of lipid molecules within the membrane, and the latter became more fluid. This was related to the substantial change in tilt angle of acyl chains from ~22° for the intact bilayer to ~35–38° after lipopeptide binding. Simple geometrical considerations led to the conclusion that such a change in tilt angle would result in ~0.6 nm thinning of the membrane. According to AFM data, the membrane thinning was found to be ~1.0 nm; however, it should be noted that tip-sample interaction during AFM imaging results in elastic deformation of the soft film [33]. Consequently, the bilayer is slightly compressed under the tip load, which in turn gives underestimated values of the thickness.

**Figure 6:**
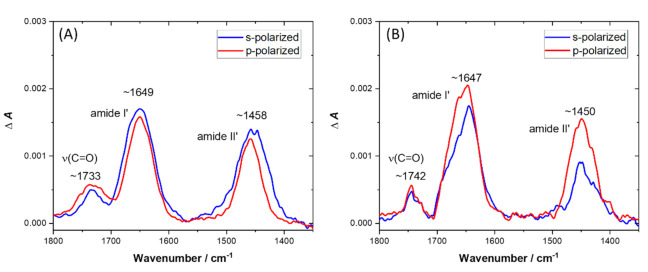
ATR-FTIR spectra in amide region recorded for DPPG/POPG/CL bilayer deposited onto the Si prisms after ~60 min of exposure to LC10-OP (**A**) and BC10-OP (**B**). The spectra were recorded in 0.01 M PBS dissolved in D_2_O. The red line corresponds to the spectrum recorded with p-polarized light, while the blue line corresponds to the spectrum recorded with s-polarized light.

Simultaneously with the increase in (C–H) bands, the emergence of a broad υ(C=O) band from a lipid ester bond was observed, accompanied by amide I’ and amide II’ bands (see Figure 6). The presence of the ester υ(C=O) band suggests that the change in tilt angle of lipid molecules indeed occurred after lipopeptide binding. However, there was a slight difference in the position of the global maximum of this band. Specifically, upon binding with LC10-OP, this maximum was located at ~1733 cm^−1^, while, after binding BC10-OP, the maximum was found at ~1742 cm^−1^. The position of the ester υ(C=O) band is known to be sensitive to the extent of hydrogen bonding and hydration of the polar head region of lipid membrane [34]. In the case of phosphatidylglycerols and cardiolipins, there are usually two components of the ester carbonyl band centered at ~1742 cm^−1^ and ~1728 cm^−1^, corresponding to dehydrated and hydrated carbonyl groups, respectively [35]. Hence, the higher frequency observed for the ester υ(C=O) band after binding of BC10-OP may indicate that the lipopeptide provided a less hydrated or less polar environment for the carbonyls compared with LC10-OP. It should be noted that the carbonyl group was in the interfacial region between the hydrophilic and hydrophobic parts of the lipid molecule. Therefore, the differences in hydration or polarity of the environment surrounding the carbonyl groups may reflect different depths of lipopeptide insertion. Specifically, the lipophilic part of BC10-OP may penetrate deeper into the hydrophobic core of the membrane. Such an interpretation seems to be reasonable if we consider that binding of BC10-OP resulted in a lower value of the order parameter and chain tilting was higher compared with LC10-OP. Further differences in the behavior of lipopeptides after binding with the lipid bilayer became apparent during the analysis of amide bands (see Figure 6). The position of the amide I’ band is sensitive to the conformation of the peptide chain [36]. For LC10-OP, the global maximum of the amide I’ band occurred at ~1649 cm^−1^, while, for BC10-OP, it was observed at ~1647 cm^−1^; however, there were visible shoulders at ~1660 cm^−1^. These values were indicative of an irregular and unordered structure of peptide moieties with a plausible contribution from β-turns. The presence of amide II’ bands located at ~1458 cm^−1^ and 1450 cm^−1^ demonstrates that deuteration of amide NH groups occurred [36]. Since the peptide moieties did not adopt any well-defined secondary structure, it was difficult to determine their orientation upon binding with lipid bilayer. However, the average tilt angles of the amide C=O transition dipole moment with respect to the surface normal were found to be 79° (±4°) and 65° (±3°) for LC10-OP and BC10-OP, respectively. Considering this divergence in conjunction with the differences in the hydration of the lipid ester group, this may indicate that the plane of amide bonds in LC10-OP was almost parallel to the plane of the bilayer, while, in BC10-OP, the peptide moiety either adopted a slightly more tilted orientation or its molecular axis was rotated, enabling deeper insertion of the lipopeptide into the membrane. Such an interpretation is in line with the results of surface pressure measurements, where more efficient insertion was observed for BC10-OP.

## 4. Conclusions

We demonstrated that both LC10-OP and BC10-OP display antimicrobial activity with the lowest values of minimum inhibitory concentrations found for Gram-positive *S. aureus* and *S. epidermidis*. Due to the amphipathic nature of the lipopeptides, the most probable target of their antimicrobial action is the cell membrane. Therefore, the mechanism of their action was evaluated on a molecular level using model lipid films composed of DPPG/POPG/CL mimicking the membrane of Gram-positive bacteria. The surface pressure measurements revealed that both lipopeptides showed the ability to bind and incorporate into the lipid monolayers. As a result, the limiting molecular area was substantially increased and the changes in compression modulus proved membrane fluidization. The same effect was observed for the DPPG/POPG/CL bilayer supported on a solid substrate. As can be concluded from AFM data, the exposure of the bilayer to lipopeptides led to a transition from the ordered gel phase to disordered liquid crystalline phase, which was manifested by ~1.0 nm thinning of the membrane. This observation correlates with the spectroscopic results. Quantitative analysis using ATR-FTIR measurements revealed that lipopeptide binding caused a substantial increase in the average tilt angle of lipid acyl chains with respect to the surface normal. This angle was changed from ~22° for the intact DPPG/POPG/CL bilayer to ~35° for LC10-OP and ~38° for BC10-OP. Spectroscopic results demonstrated also that peptide moieties in both molecules did not adopt any well-defined secondary structure upon binding with the lipid membrane. Interestingly, the lipid films were noticeably more affected by BC10-OP, which seemed to insert deeper into the DPPG/POPG/CL membrane compared with LC10-OP. Since the peptide motifs are the same in both lipopeptides, the observed effect can be ascribed to the small difference in structure of the lipophilic chain, which alters the balance between the hydrophobic and hydrophilic portions of the molecules.

## Figures and Tables

**Table 1:** Minimum inhibitory concentrations (MICs) determined for lipopeptides with a linear decanoyl chain (LC10-OP) or with a branched 4-methylnonanoyl chain (BC10-OP). PCM, Polish Collection of Microorganisms; ATCC, American Type Culture Collection.

Strain	MIC (mg/L)	
	LC10-OP	BC10-OP
(+) *Enterococcus faecalis* ATCC 14506	n.d.	n.d.
(–) *Klebsiella pneumoniae* PCM 1	n.d.	30
(–) *Pseudomonas aeruginosa* PAO1 PCM 499	20	20
(+) *Staphylococcus aureus* ATCC 29213	10	10
(+) *Staphylococcus epidermidis* ATCC 12228	5	10
(–) *Yersinia enterocolitica* PCM 2081	n.d.	n.d.

n.d.—not determined, i.e., the MIC was higher than 50 mg/L.

**Table 2:** Characteristic parameters of Langmuir monolayers.

Subphase	Limiting Molecular Area (Å^2^)	Maximum *C*_s_^−1^ (mN/m)
**Monolayer: DPPG/POPG/CL (1:1:2)**		
0.01 M PBS	76.0 ± 0.4	139 ± 7
0.1 μM *CL10-OP*	84.0 ± 2.8	145 ± 8
0.5 μM *CL10-OP*	123.9 ± 2.4	97 ± 6
1.0 μM *CL10-OP*	141.5 ± 1,3	70 ± 4
**Monolayer: DPPG/POPG/CL (1:1:2)**		
0.01 M PBS	76.0 ± 0.4	139 ± 7
0.1 μM *BL10-OP*	96.8 ± 0.6	138 ± 6
0.5 μM *BL10-OP*	151.0 ± 2.0	77 ± 4
1.0 μM *BL10-OP*	181.1 ± 0.8	54 ± 10

**Table 3:** Molecular ordering and orientation data ^a^.

Lipid Bilayer	Dichroic Ratio (*R*)	*S*_chain_	*θ*_chain_ (°)
DPPG/POPG/CL	0.96 ± 0.02	0.78 ± 0.03	22 ± 2
DPPG/POPG/CL + *LC10-OP*	1.14 ± 0.04	0.50 ± 0.06	35 ± 2
DPPG/POPG/CL + *BC10-OP*	1.18 ± 0.03	0.43 ± 0.04	38 ± 1

^a^ The reported parameters were determined from υ_s_(CH_2_) absorption bands.

## Data Availability

Not applicable.
